# Dysbiosis of Cervical and Vaginal Microbiota Associated With Cervical Intraepithelial Neoplasia

**DOI:** 10.3389/fcimb.2022.767693

**Published:** 2022-02-14

**Authors:** Suibin Lin, Bin Zhang, Yixia Lin, Yueping Lin, Xiaoyu Zuo

**Affiliations:** ^1^ Department of Obstetrics & Gynecology, Zhangpu Hospital, Zhangzhou, China; ^2^ Department of Obstetrics and Gynecology, The First Affiliated Hospital of Fujian Medical University, Fuzhou, China; ^3^ Department of Pediatric Surgery, Guangzhou Institute of Pediatrics, Guangdong Provincial Key Laboratory of Research in Structural Birth Defect Disease, Guangzhou Women and Children’s Medical Center, Guangzhou Medical University, Guangzhou, China

**Keywords:** cervical intraepithelial neoplasia, dysbiosis, cervicovaginal microbiota, 16S rRNA, microbial migration

## Abstract

Cervical intraepithelial neoplasia (CIN) is a precancerous condition inducing local lesions on the surface of the squamocolumnar junction of the cervix. Despite the role of vaginal microbiota having been under-discussed, the role of the cervical microbiome and the microbial migration across the reproductive tract involved in CIN was limitedly studied. We aimed to synchronously characterize the dysbiosis associated with CIN in both the cervix and vagina in a Chinese population. Profiling of cervical and vaginal microbiota from 60 CIN women and 60 healthy women was conducted. 16S rRNA sequencing was adopted. By comparing the microbial profiles between different parts of the reproductive tract, our results demonstrated an increased shift of microbial diversity in the cervix compared with that in the vagina for the CIN patients, specifically in CIN 1. Less dysbiosis was found between the CIN patients and controls, in either the vagina or cervix. The microbial community may be modulated by the onset of sexual activity, a known clinical risk factor for cervical neoplasia. Distinct patterns of perturbated bacteria were found in the vaginal and cervical microbiota, in which reduced Actinobacteria-related operational taxonomic units (OTUs) and increased Proteobacteria-related OTUs were found in the vagina and cervix, respectively. A good agreement between the direction of the top-significant perturbated OTUs was observed between the vaginal and cervical microbiome, suggesting a potential microbial migration in the reproductive tract. Enriched genera such as *Sphingomonas* and *Stenotrophomonas* were found in cervical microbiota-associated CIN. Multivariate analysis revealed *Comamonas*, *Rhizobium*, and *Pseudomonas* as independent genera contributing to CIN in the cervix. In summary, this study revealed the perturbation of microbiota in the presence of CIN and demonstrated a distinct pattern of characteristic bacteria community between the vagina and cervix involved in the development of CIN.

## Introduction

Cervical intraepithelial neoplasia (CIN) is a precancerous condition featured by abnormal growth of cells on the surface of the squamocolumnar junction of the cervix. According to the degree of affected tissue, CIN can be classified as CIN 1 (low-grade neoplasia), CIN 2, and CIN 3 (the most severe form). Although low-grade dysplasia (CIN 1) can regress, it may progress to high-grade dysplasia (CIN 3) and, even worse, to cervical carcinoma. Known risk factors for CIN include early onset of sexual activity, multiple sex partners, cigarette smoking, sexually transmitted virus infection, and history of vulva and anus dysplasia (Wipperman et al., 2018). Persistent infection by human papillomaviruses (HPVs), especially the high-risk HPV-16 and HPV-18 subtypes, is the primary risk factor involved in the development of CIN (Steenbergen et al., 2014). HPV can induce altered proliferation and differentiation of invaded cells and promote malignant conversion *via* viral proteins E6 and E7 (Hoppe-Seyler et al., 2018; Carrero et al., 2021). Besides, HPV could induce local lesions by impairing the innate and adaptive immunity of cervical mucosa (Khoury et al., 2018; Britto et al., 2020) and impair the normal antibacterial microenvironment of the reproductive tract (Hoffman et al., 2017; Szymonowicz and Chen, 2020).

The commensal microbial community is benefited in maintaining homeostasis, modulating the host immune system and metabolism (Zheng et al., 2020). Perturbation of microbial community, known as dysbiosis, contributes in many human complex diseases such as metabolic disease, gastrointestinal disease, and carcinoma. Considered as an environmental factor, a microbial community could produce more than four million products to induce metabolic infection in low-grade inflammation characterizing metabolic disease (Burcelin, 2012; Tilg et al., 2020). Microbiota perturbation in the reproductive tract has been associated with gynecological cancers (Mert et al., 2018). Increased abundance of *Atopobium vaginae* and *Porphyromonas* spp. was found in women with cancer (Walther-Antonio et al., 2016).

The role of the cervicovaginal microbiome on the development of CIN has been discussed (Curty et al., 2019). A notable characteristic is the increased diversity of the vaginal microbiota (Castanheira et al., 2021) and cervical microbiota (Audirac-Chalifour et al., 2016) in cervical neoplasia. Vaginal microbiota composition may be a promising biomarker to predict the CIN progression (Curty et al., 2019; Mitra et al., 2020). Excessive genera such as *Gardnerella* and reduced *Lactobacillus* were revealed in CIN (Mitra et al., 2020; Kang et al., 2021). Similarly, increased microbial diversity and decreased levels of *Lactobacillus* spp. were prevalent in the CIN patients (Mitra et al., 2015). Emerging studies indicated that the cervicovaginal microbiota plays a crucial role in HPV persistence and contributes to the development of premalignant lesions (Brotman et al., 2014; Hoppe-Seyler et al., 2018; Ilhan et al., 2019; Wiik et al., 2019; Usyk et al., 2020). A longitudinal study suggested that *Lactobacillus gasseri*-dominant communities may help the clearance of acute HPV infection (Brotman et al., 2014). Metabolic profiling indicated many inflammatory and metabolic processes crucial for HPV infection, and persistence was promoted by the cervicovaginal microbiota (Ilhan et al., 2019). Although the role of the vaginal microbiota is well studied (Castanheira et al., 2021), microbiota in the upper reproductive tract such as the cervix was not fully explored (Agostinis et al., 2019). *Sneathia* spp. and *Fusobacterium* spp. were found to be predominant in the intraepithelial lesion and cervical cancer, respectively, in Mexican women (Audirac-Chalifour et al., 2016). Zhang et al. suggested that the effect of the cervical microbiota on CIN progression was mediated by HPV infection (Zhang et al., 2018). Pathway analysis pointed out that folate biosynthesis and oxidative phosphorylation were pronounced in high-grade CIN (Tango et al., 2020). However, the relationship between the vaginal and cervical microbiota and their role in the risk of CIN was limitedly explored.

In this study, we collected both cervical and vaginal specimens from 60 women under the condition of CIN, and we conducted microbial profiling of the reproductive tract of 60 healthy women with matched age and district. By comparing the microbial profiles between different parts of the reproductive tract, we revealed the perturbation of microbial community in the presence of CIN and found microbial translocation in the reproductive tract that was linked with CIN development.

## Materials and Methods

### Study Participants

This study was approved by the Ethical Committee of the Zhangpu Hospital (Fujian province, China) and was performed according to principles stated in the Declaration of Helsinki. A total of 60 women diagnosed with CIN by histopathology were recruited in this study. Sixty age- and district-matched healthy participants were recruited from the physical examination center of Zhangpu Hospital as the control group. All healthy participants were excluded from CIN by Pap smear and colposcopy examination. Clinical characteristics such as body mass index (BMI), sexual activity, and sex partner were collected. Written informed consent was obtained from each participant.

### Specimen Collection and DNA Extraction

Both cervical and middle vaginal specimens were collected for each participant. For each participant undergoing vaginal endoscopy, cotton swabs were gently rotated about 10 s to adequately collect microbial specimens located in the middle-to-upper vagina and the cervix. To avoid contamination by microbes of the lower vagina, the cotton swab was placed inside a tube to isolate it from the human body. The specimens were placed in a sterilized container and either kept at 4°C for a maximum of 4 h before being processed or immediately frozen and stored at −80°C until further use. The DNA was extracted using a QIAamp DNA Mini Kit (Qiagen, Valencia, CA, USA) according to the manufacturer’s protocol. DNA quality was assessed by Agilent Bioanalyzer 2100 (Agilent Technologies, Santa Clara, CA, USA) and a Qubit fluorometer (Life, Waltham, MA, USA).

### 16S rRNA Gene Sequencing

For bacterial profiling, a region-specific primer (338F-806R) targeting the V3–V4 variable regions of the 16S rRNA gene was used for PCR amplification. The primer pairs were 5′-ACTCCTACGGGAGGCAGCA-3′ (forward) and 5′-GGACTACHVGGGTWTCTAAT-3′ (reverse). Libraries were prepared by using TruSeq Nano DNA LT Library Prep Kit (FC-121-4001, Illumina, San Diego, CA, USA) and purified by using AMPure XP purification beads (Beckman Coulter, Brea, CA, USA). The amplicon library was quality evaluated by an Agilent Bioanalyzer 2100 (Agilent Technologies, Santa Clara, CA, USA) and quantified by a Promega (Madison, WI, USA) QuantiFluor. Libraries with unimodal peak distribution and sufficient concentration (>2 nM) were pooled and sequenced on a MiSeq platform (Illumina, San Diego, CA, USA) to generate 2 × 300 bp of paired-end reads.

### 16S rRNA Amplicon Sequence Analysis

Raw sequencing data were filtered based on sequencing quality using Trimmomatic software. Clean read pairs were merged into fragments according to the overlaps between reads and their mates. Next, merged sequences were clustered into operational taxonomic units (OTUs) using UNOISE algorithm with a 97% identity threshold by using the VSEARCH program. Taxonomical classification of OTUs was conducted by the UCLUST algorithm in QIIME (V1.9) based on the EzBioCloud reference database (Version 2017.10). Low abundant OTUs (<0.01% relative abundance) were filtered out. The OTU table was subjected to scale normalization by scaling the sequencing depth to a fixed value of 20,000 sequences using the VSEARCH program. Phylogeny tree for the normalized OTUs was built using the FastTree algorithm implemented in QIIME software. Rarefied alpha diversity was evaluated by a rarefaction curve that steps from 500 to 20,000 sequence depth. The alpha diversity was calculated using the normalized OTU table. The pairwise Bray–Curtis (BC) distance was calculated to investigate the similarity of the microbial communities between different groups. Relative abundances of different taxonomic levels (phylum, class, order, family, and genus) were summarized based on a normalized OTU table using summarize_taxa.py script in QIIME.

### Statistical Analysis

Statistical analyses and data visualization were performed in R (v3.6). Principal coordination analysis (PCoA) followed by permutational multivariate ANOVA (PERMANOVA) was used to evaluate the beta diversity. Relative abundance was subjected to arcsine-squared transformation for visualization convenience. Wilcoxon rank-sum test was used to detect differential abundant OTU and taxa. Multivariate analysis with linear models (MaAsLin) was also applied to correct for covariates and identify the differentially abundant taxa. We used the Q-value package implemented in MaAsLin analysis. False discovery rate (FDR) was used for multiple testing correction of *p*-value. Spearman’s coefficient was used for correlation analysis. An FDR of 0.05 was used as the cutoff value for significance.

## Results

### Subject Characteristics

A total of 60 women diagnosed with CIN (denoted as CIN) and 60 age-matched and district-matched healthy women [denoted as healthy control (HC)] were included in this study (**Table 1**). The median age for the CIN group and control group were 40.00 and 38.00, respectively. No significant difference was found for the BMI (*p* = 0.198), onset of sexual activity (*p* = 0.064), and the proportion of multiple sex partners (*p* = 0.741). A significantly higher HPV-positive rate was observed in the CIN group compared with controls (89.5% vs. 57.9%, *p* < 0.001). Among the CIN patients, there were 68.3% patients classified as CIN 1, 10% as CIN 2, and 21.7% as CIN 3.

**Table 1:** Baseline characteristics of the study subjects.

	Control (*n* = 60)	CIN (*n* = 60)	*p*
Age (year), median (Q1–Q3)	38.00 (29.75–49.00)	40.00 (30.75–47.25)	0.483
BMI (mean ± SD)	22.55 ± 3.95	21.75 ± 2.80	0.198
Onset of sexual activity (year), median (Q1–Q3)	22.00 (20.00–24.00)	21.50 (19.00–23.00)	0.064
Number of sex partners, *n* (%)			
1	54 (90.0)	56 (93.3)	0.741
>1	6 (10.0)	4 (6.7)	
HPV-positive, *n* (%)	33 (57.9)	51 (89.5)	<0.001
CIN grade, *n* (%)			
CIN 1	N/A	41 (68.3)	N/A
CIN 2	N/A	6 (10.0)	
CIN 3	N/A	13 (21.7)	

Q1, the first quantile; Q3, the third quantile; SD, Standard Deviation; CIN, cervical intraepithelial neoplasia; BMI, body mass index; CIN, cervical intraepithelial neoplasia; HPV, human papillomavirus.

N/A means data inapplicable.

### Dysbiosis of Cervical Microbiota Associated With Cervical Intraepithelial Neoplasia

**Figure 1:**
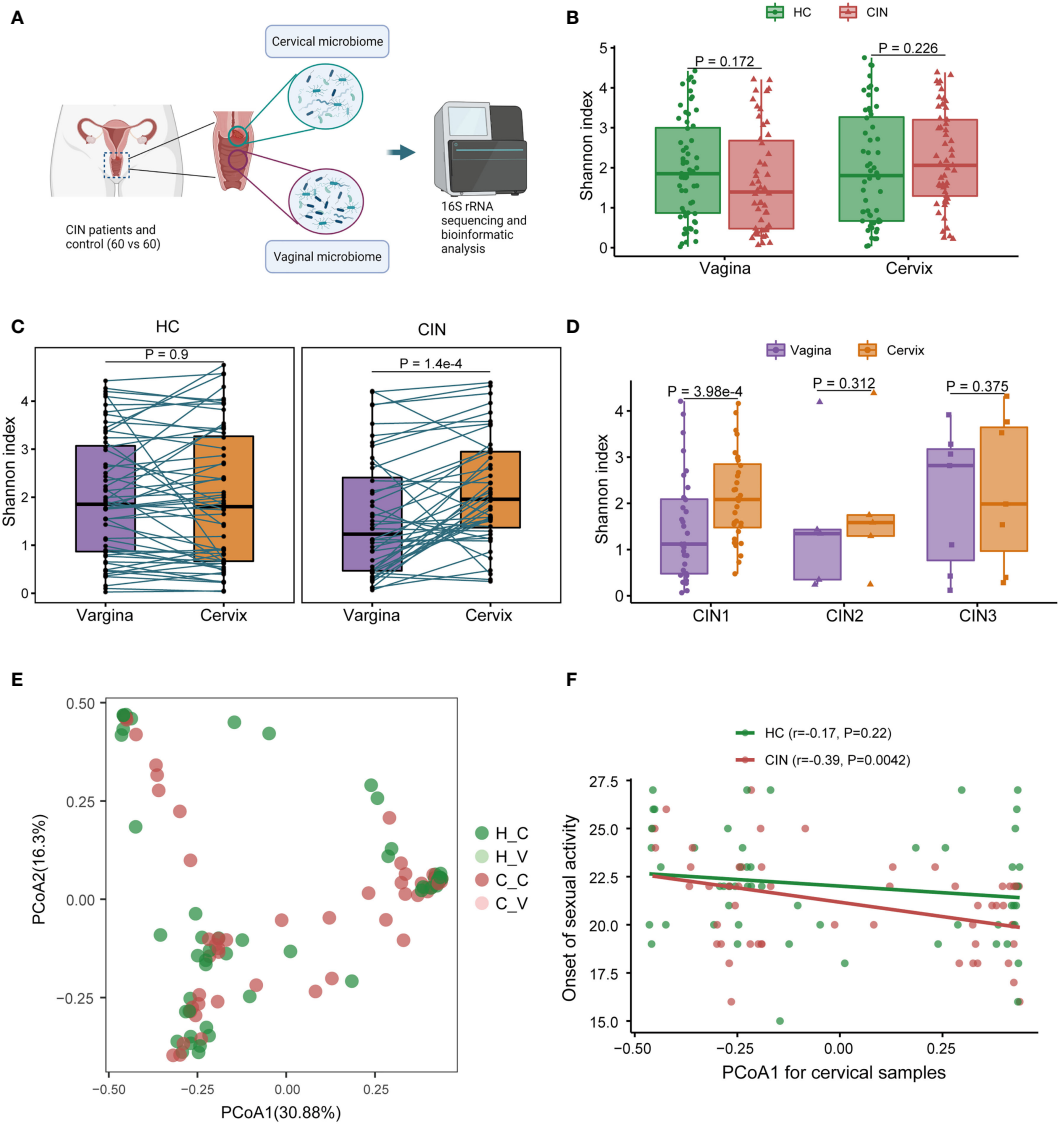
Vaginal and cervical microbial diversity change associated with cervical intraepithelial neoplasia (CIN). **(A)** Schema for the study design. **(B)** Microbial diversity associated with CIN in the vagina and cervix. **(C)** Comparison of microbial diversity across the vagina and cervix. **(D)** Microbial diversity between the vagina and cervix in different CIN subtypes. **(E)** Principal coordination analysis (PCoA) result for microbial community. **(F)** Correlation between PCoA1 and onset of sexual activity in the cervix.

For each participant in the CIN patients (CIN) and HC, both cervical and middle-upper vaginal specimens were collected for pairwise comparison of microbiome change across women’s reproductive tract. The schema for the study design is illustrated in **Figure 1A**. After quality control, 4 specimens in HC and 15 specimens in CIN were filtered. We first observed the variation of microbial diversity between CIN and HC across the vagina and cervix (**Figure 1B**). Unfortunately, no significant changes in Shannon diversities were found in CIN (*p* = 0.172 in the vagina and *p* = 0.226 in the cervix; **Figure 1B**), despite that the observed OTU counts for CIN were marginally greater than those for HC in the cervical specimen (mean count: 71.9 vs. 58.3, *p* = 0.031). However, the microbial diversity was increased in cervical specimens against their paired vaginal specimens in CIN (*p* = 1.4 × 10^−4^, **Figure 1C**). Moreover, subtype analysis of CIN revealed the pronounced increase of Shannon diversity in the cervical microbiome in the CIN 1 subtype (*p* = 3.98 × 10^−4^, **Figure 1D**).

To characterize the vaginal and cervical microbial community in CIN, we next calculated the BC distance between each specimen according to the OTU level abundance (**Figure 1E**). Under the present sample size setting, no obvious community dissimilarities were found between HC and CIN across the vagina or cervix (adenosis, *p* = 0.368, **Figure 1E**). Further exploration on the relationship of the first component of PCoA (PCoA1) with the clinical variables identified a significant negative correlation between PCoA1 and the onset of sexual activity in the cervical microbiome in CIN (Spearman’s *r* = −0.39, *p* = 0.0042, **Figure 1F**). Taken together, our results demonstrated an increased shift of microbial diversity in the cervix for the CIN patients and suggested that the cervical microbial community may be modulated by the clinical risk factor in CIN.

### Identification of Characteristic Bacteria Involved in Cervical Intraepithelial Neoplasia

**Figure 2:**
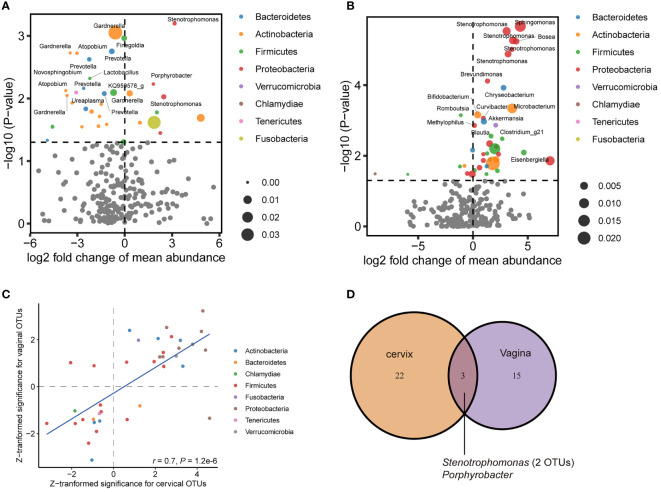
Differential abundance of operational taxonomic unit (OTU) associated with cervical intraepithelial neoplasia (CIN) in the vagina and cervix. **(A)** Volcano plot showing perturbated OTUs in the vagina. **(B)** Volcano plot showing perturbated OTUs in the cervix. **(C)** Correlation of Z-transformed significance of top significant OTUs between the vagina and cervix. **(D)** Venn diagram showing shared significant (*p* < 0.01) OTUs associated with CIN between the vagina and cervix.

To further characterize the perturbated microbiota associated with CIN, we first estimated the prevalence of all bacteria detected in our samples (**Supplementary Table 1**). The prevalence of OTUs annotated to the same genus was summed to estimate the prevalence of that genus. We then examined the differential abundances of OTUs and existing taxa in consecutive levels (e.g., phylum, family, and genus). The results showed that for women with CIN, the differential abundance OTUs for the cervical microbiome had a distinct pattern against that for the vaginal microbiome. While reduced abundance for Actinobacteria-related OTUs was pronounced to be associated with CIN in the vaginal specimen (**Figure 2A**), enriched Proteobacteria-related OTUs featured the CIN-associated dysbiosis in the cervical specimen (**Figure 2B**). The *Lactobacillus*-related OTUs were decreased, whereas, interestingly, a *Gardnerella*-related OTU was significantly reduced in the vagina (log2-fold change (log2FC) = −0.60, *p* = 0.0009, **Supplementary Table 1**). By comparison of the top-ranked OTUs in both the vagina and cervix, we found a very good agreement between the direction of variation between the two locations (Spearman’s *r* = 0.7, *p* = 1.20 × 10^−6^, **Figure 2C**), in which Proteobacteria-related OTUs were consistently increased in CIN. A representative genus for these OTUs is *Stenotrophomonas* with log2FC of 3.55 (*p* = 1.20 × 10^−6^) and 3.16 (*p* = 6.32 × 10^−4^) in the cervix and vagina, respectively (**Supplementary Table 2**). However, only 3 OTUs (2 from *Stenotrophomonas* and 1 from *Porphyrobacter*) were consistently significant in both the vagina and cervix (*p* < 0.01, **Figure 2D**), suggesting the independent signatures for the respective location.

**Figure 3:**
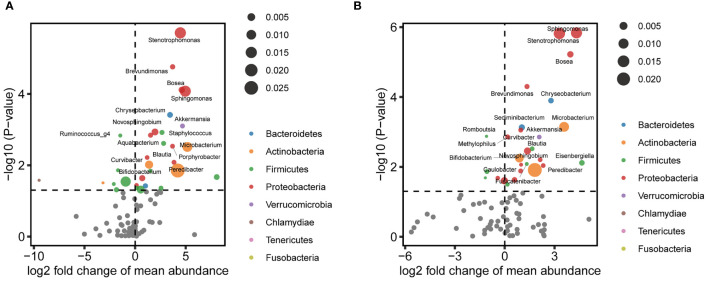
Differential abundance of genera associated with cervical intraepithelial neoplasia (CIN) in the vagina and cervix. **(A)** Volcano plot showing perturbated genera in the vagina. **(B)** Volcano plot showing perturbated genera in the cervix.

Next, representative microbial taxa were identified for the alteration of the vaginal and cervical microbiota associated with CIN. OTUs belonging to the same taxon were aggregated. Like the OTU-level results, we observed only a limited number of differential abundance genus in the vagina. While genera *Finegoldia*, *Prevotella*, and *KQ959578_g* (a genus of Ruminococcaceae) were negatively correlated with CIN, the *Porphyrobacter*, *Blautia*, and *Rhodococcus* were upregulated in CIN (**Figure 3A** and **Supplementary Table 3**). Likewise, a significantly higher abundance of *Sphingomonas*, *Stenotrophomonas*, *Bosea*, *Brevundimonas*, and *Chryseobacterium* was identified to be associated with CIN in the cervical microbiota (**Figure 3B** and **Supplementary Table 4**). We further performed the MaAsLin to adjust the potential covariables such as age, BMI, and HPV infection. MaAsLin identified *Comamonas*, *Rhizobium*, and *Pseudomonas* as the remarkably leading bacteria associated with CIN in the cervix (**Table 2** and **Supplementary Table 5**).

**Table 2:** Differential abundance genus associated with CIN in cervix detected by multivariate analysis.

Phylum	Class	Order	Family	Genus	HC^a^	CIN^a^	log2FC	*p*	*p_m_*	*p_madj_*
Proteobacteria	Betaproteobacteria	Burkholderiales	Comamonadaceae	*Comamonas*	0.38	0.38	−0.02	2.50E−02	2.58E−05	5.59E−03
Proteobacteria	Alphaproteobacteria	Rhizobiales	Rhizobiaceae	*Rhizobium*	0.07	0.13	0.86	3.04E−01	3.78E−05	5.59E−03
Proteobacteria	Gammaproteobacteria	Pseudomonadales	Pseudomonadaceae	*Pseudomonas*	0.17	0.89	2.39	4.46E−01	6.61E−04	4.89E−02
Proteobacteria	Alphaproteobacteria	Caulobacterales	Caulobacteraceae	*Brevundimonas*	0.04	0.10	1.34	5.02E−05	1.02E−03	5.02E−02
Proteobacteria	Betaproteobacteria	Methylophilales	Methylophilaceae	*Methylophilus*	0.08	0.09	0.17	1.39E−03	2.10E−03	8.76E−02
Proteobacteria	Betaproteobacteria	Burkholderiales	Ralstonia_f	*Ralstonia*	0.13	0.02	−2.91	5.75E−01	2.37E−03	8.76E−02
Proteobacteria	Betaproteobacteria	Burkholderiales	Comamonadaceae	*Curvibacter*	0.04	0.08	0.96	8.86E−04	3.71E−03	1.00E−01
Proteobacteria	Betaproteobacteria	Methylophilales	Methylophilaceae	Other	0.02	0.02	0.17	1.03E−01	3.72E−03	1.00E−01
Proteobacteria	Betaproteobacteria	Burkholderiales	Comamonadaceae	*Aquabacterium*	0.05	0.09	0.96	1.31E−02	4.80E−03	1.09E−01
Proteobacteria	Deltaproteobacteria	Bdellovibrionales	Bacteriovoracaceae	*Peredibacter*	0.01	0.07	2.32	9.16E−03	7.68E−03	1.51E−01
Proteobacteria	Gammaproteobacteria	Pseudomonadales	Moraxellaceae	*Enhydrobacter*	0.05	0.02	−1.02	5.45E−01	9.37E−03	1.70E−01
Fusobacteria	Fusobacteria_c	Fusobacteriales	Fusobacteriaceae	*Fusobacterium*	0.03	0.03	0.17	5.13E−01	9.75E−03	1.70E−01
Firmicutes	Bacilli	Lactobacillales	Aerococcaceae	*Granulicatella*	0.02	0.03	0.69	9.04E−02	1.46E−02	2.41E−01

p, univariate p-values derived from Wilcoxon rank-sum test; p_m_, multivariate p-values derived from MaAsLin, adjusting for age, BMI, onset of sexual activity, and HPV infection; p_mad_, FDR-corrected multivariate p-values to account for multiple testing.

CIN, cervical intraepithelial neoplasia; HC, healthy control; MaAsLin, multivariate analysis with linear models; BMI, body mass index; HPV, human papillomavirus; FDR, false discovery rate.

a Mean relative abundance of genus in HC and CIN in terms of percentage (%).

## Discussion

In this study, we performed microbial profiling for both cervical and vaginal microbiome associated with CIN. By comparing the microbial profiles between different parts of the reproductive tract, we revealed the perturbation of microbial community in the presence of CIN and found the suspected microbial translocation in the reproductive tract that was linked with CIN. Our results demonstrated an increased shift of microbial diversity in the cervix compared with that in the vagina for the CIN patients. More specifically, dysbiosis was pronounced in CIN 1. However, less dysbiosis was found between the CIN patients and controls, in either the vagina or cervix. The microbial community appeared to have two distinguished clusters, suggesting that there exist unrevealed driving factors or the heterogeneous nature of the disease. For example, our results suggested that the microbial community may be modulated by the onset of sexual activity, which is well known as a clinical risk factor for cervical neoplasia. We further turned to pinpoint the perturbated bacteria associated with CIN in the vagina and cervix. Distinct patterns of perturbated bacteria were found in the vaginal and cervical microbiota. Reduced Actinobacteria-related OTUs and increased Proteobacteria-related OTUs were found in the vagina and cervix, respectively, suggesting that depleted “normal” bacteria in the vagina and enriched “abnormal” bacteria in the cervix characterize the dysbiosis during the development of CIN.

The identification of CIN-associated bacteria could have potential clinical benefits. One applicable approach is the non-invasive early screening for CIN in the general population. A combined quantitation of the enriched and depleted microbes such as the “dysbiosis index” (AlShawaqfeh et al., 2017) may have high sensitivity for detecting CIN. Moreover, given the advantage of this study that leveraged the vertical dysbiosis of the female reproductive tract, it is possible to extend the “dysbiosis index” to a two-way dysbiosis index that jointly accounts for the change of position and quantity. Another interesting application is to modulate the vaginal and cervical microbial communities using pre- and probiotics (Mitra et al., 2016). The usage of probiotics to regulate the cervicovaginal microbial community may be able to prevent or intervene in the progression of CIN to a certain extent. However, there is a long way to go since the preclinical assessment of safety and efficacy needs to be assessed (Fernandez-Romero et al., 2015).

Migration or translocation of microbiota in the reproductive tract has been proposed. Ascending infection is the most common route of deleterious microbes to cause upper genital infection (Kim et al., 2015). Commonly migrated microbes including *Gardnerella vaginalis* are discussed in a recent review (Cappelletti et al., 2020). The mechanism behind ascending migration has not been fully understood. Decrease of the protective *Lactobacillus* causes elevated pH, resulting in an impaired barrier. The latter facilitates the localization and proliferation of adverse bacteria such as *Gardenella* and *Prevotella* to cause dysbiosis and CIN. However, more evidence is required to elucidate this assumption. In this study, we found a good agreement between the direction of the top-significant perturbated OTUs associated with CIN between the vaginal and cervical microbiome and observed consistently overrepresentation of Proteobacteria-derived OTUs in CIN. Although *Lactobacillus* was apparently reduced in the cervix of the CIN patients, no statistical significance was observed under the current sample size setting. On the other hand, we should be aware that contamination across the cervix and vagina is hard to be eliminated in the real world. Although we used a tube to isolate the swab, our study is still at risk of cross-contamination of microbes in the vagina and cervix. Thus, the explanation of the present results should be cautious.

HPV infection is well recognized as a disease-causing microbe involved in CIN. In addition to the direct pro-tumor effect through impairing the innate and adaptive immunity of cervical mucosa, HPV may play potential roles in mediating the commensal cervicovaginal bacteria, and vice versa. Emerging studies indicated the importance of the cervicovaginal microbiota in maintaining HPV persistence and the subsequent premalignant lesions (Brusselaers et al., 2019). Thus, we also examine the relationships between the cervicovaginal microbiota and HPV infection in patients with CIN. Unluckily, the only limited difference in microbiota was found to be associated with HPV infection. We did not observe altered microbial diversity associated with HPV infection. But as a clue, we found the abundance of Lachnospiraceae EU728721_g decreased in the cervical microbiota in HPV-positive women (MaAsLin effect size −3.72, *p* = 0.00055, **Supplementary Table 5**). Currently, it is not clear how HPV and the associated predominant cervical microbiome interplay to progress the development of CIN. One possible hypothesis is that the alteration of the cervical microbiome, e.g., reduced beneficial bacteria, impairs the host’s innate immunity and predisposes the mucosa to damage by HPV infection. In turn, the damage-induced inflammatory and immune response caused by HPV infection may further perturb the microbial community. However, we recognized that the present study could not clarify causality from the observed associations. Due to the heterogeneity in the CIN patients that may be associated with the confounding factors including a number of sexual partners, onset of sexual activity, or smoking, it is difficult to directly elucidate the relationship between HPV infection and the cervicovaginal microbiota, and the underlying immunologic mechanism. A careful longitudinal monitoring design for the same individual patients might be a good solution to overcome this limitation (Usyk et al., 2020).

The current study has several limitations. First, 16S rRNA sequencing is not an unbiased estimate of microbial community due to the preference for PCR amplification. 16S rRNA sequencing has the advantage of low cost and computational efficiency on profiling and is suitable for the exploration of the microbiome for large-scale cohorts. Further metagenomics study is required to comprehensively study the microbial community and determine the responsible species and strain at a higher resolution and confidence. Second, only microbiome associations were evaluated in this study such that a great proportion of underlying genetic susceptibility-dysbiosis interplay was missed. Host genetics has been demonstrated to mediate the alteration of gut microbiota, such as cardiovascular disease (Zhernakova et al., 2018) and inflammatory bowel disease (Knights et al., 2014). In addition, given the diverse lifestyle and cultures in different regions of China, the microbial profiling in this study may not fully represent the entire Chinese population. The relationship between genetic background, geography, life cultures, and their interplays with microbiome across the subpopulations in China is a complicated but interesting topic. It is advisable to take host genetics and multiregion under consideration for the follow-up study. Third, the sample size is relatively limited in this study, resulting in the relatively insufficient power to identify the CIN-associated bacteria with a weak effect size. Although our results were biologically meaningful and supported by previous studies, we acknowledge that future large-scale studies are warranted to validate the present findings.

In summary, the present study can serve as a good start point to narrow down the candidate pathogens for future assays and provide new evidence for the microbiome in women’s reproductive tract associated with CIN.

## Data Availability Statement

The raw sequence data reported in this paper have been deposited in the Genome Sequence Archive in National Genomics Data Center, China National Center for Bioinformation / Beijing Institute of Genomics, Chinese Academy of Sciences (GSA: CRA005993) that are publicly accessible at https://ngdc.cncb.ac.cn/gsa.

## Ethics Statement

The studies involving human participants were reviewed and approved by the Ethical Committee of the Zhangpu Hospital (Fujian province, China). The patients/participants provided their written informed consent to participate in this study.

## Author Contributions

SL and XZ conceived the study. BZ, YxL, and YpL collected the samples and conducted the experiments. BZ and XZ participated in the data analysis and interpretation. SL and XZ drafted the manuscript. All authors read and approved the final manuscript.

## Funding

This work was supported by the subsidies of the outstanding young and middle-aged medical experts of Zhangzhou city, the National Natural Science Foundation of China (grant no. 81602478), and the Natural Science Foundation of Guangdong Province of China (grant no. 2016A030310194).

## Conflict of Interest

The authors declare that the research was conducted in the absence of any commercial or financial relationships that could be construed as a potential conflict of interest.

## Publisher’s Note

All claims expressed in this article are solely those of the authors and do not necessarily represent those of their affiliated organizations, or those of the publisher, the editors and the reviewers. Any product that may be evaluated in this article, or claim that may be made by its manufacturer, is not guaranteed or endorsed by the publisher.
